# Fatty Acids and Nutraceutical Properties of Lipids in Fallow Deer (*Dama dama*) Meat Produced in Organic and Conventional Farming Systems

**DOI:** 10.3390/foods10102290

**Published:** 2021-09-27

**Authors:** Janusz Kilar, Anna Kasprzyk

**Affiliations:** 1 Jan Grodek State University in Sanok, Institute of Agricultural and Forest Economy, 21 Mickiewicza, 38-500 Sanok, Poland; janusz.kilar@wp.pl; 2 Institute of Animal Breeding and Biodiversity Conservation, University of Life Sciences in Lublin, 13 Akademicka, 20-950 Lublin, Poland

**Keywords:** venison, feeding system, muscles, intramuscular fat, cholesterol, fatty acids

## Abstract

The aim of the study was to assess the fatty acid profile and nutraceutical properties of lipids contained in fallow deer (*Dama dama*) meat produced in organic and conventional farming systems. *Longissimus lumborum* (LL) and *semimembranosus* (SM) muscles from 24 fallow deer carcasses were selected for the study. The fallow deer meat from the organic farming system was characterized by significantly lower intramuscular fat content. The fatty acid profile in the organic meat was characterized by a particularly high proportion (*p* < 0.0001) of conjugated linoleic acid—CLA (LL—2.29%, SM—2.14%), alpha-linolenic acid—ALA (LL—4.32%, SM—3.87%), and docosahexaenoic acid—DHA (LL—2.83%, SM—2.60%). The organic system had a beneficial effect (*p* < 0.0001) on the amount of polyunsaturated fatty acids (PUFAs), including n-3 PUFAs, which resulted in a more favorable n-6 PUFA (polyunsaturated fatty acid)/n-3 PUFA ratio. The significantly higher nutritional quality of organic meat lipids was confirmed by such nutraceutical indicators as the thrombogenic index (TI), ∆9-desaturase C16, elongase, and docosahexaenoic acid+eicosapentaenoic acid (DHA+EPA) in the LL and SM and cholesterol index (CI), and the cholesterol-saturated fat index (CSI) indices in the SM. LL was characterized by higher overall quality.

## 1. Introduction

The dynamic changes in nutrition in terms of food quality and health safety requirements prompt the consumer to look for food produced in strictly defined systems, e.g., in the organic farming system [1,2]. This is confirmed by the continuous increase in the global organic food market [3,4,5] and the frequency of purchase of such products [6]. There is a common belief that organic food has higher nutritional values, is healthier and safer [7,8,9,10], and reduces the risk of overweight and obesity [11]. Recent literature reviews and meta-analyses have revealed significant differences in the nutritional composition of organic and conventional food [5,8]. Organic meat has better nutritional properties than industrially produced meat, which is reflected in a considerably improved composition of animal fats (higher concentrations of n-3 fatty acids), higher content of biologically active compounds, and lower cholesterol levels [1,12]. A higher frequency of consumption of organic meat in the diet can significantly reduce the prevalence of various lifestyle diseases [13].

Meat from game animals is a highly valued culinary and processing raw material due to its flavor, excellent nutritional properties, and high pH stability in the maturation process [14,15,16]. It can be classified as very lean meat [16,17,18]. Over the last few years, the interest in this meat has been increasing as it is regarded as “natural and sustainable” [19]. Wild-living animals experience a high standard of welfare and eat mainly natural food. Their meat is free of antibiotics and hormones [20]. Meat of various cervid species is also produced on farms. In Poland, the red deer (*Cervus elaphus*), the sika deer (*Cervus nippon*), and especially the fallow deer (*Dama dama*) are reared quite commonly [21]. Compared with other cervid species, fallow deer meat is regarded as a healthier product due to the higher content of n-3 polyunsaturated fatty acids (PUFAs) [17]. It is high in protein, heme iron, copper, zinc, and potassium and low in saturated fatty acids [17,18,22,23,24,25]. The meat of cervids is also a good source of nutritionally important conjugated linoleic acid (CLA) [25]. Due to the essential role of fat in the human diet, the quantity and quality of lipids in meat from various animal species are the focus of considerable research interest [7,26,27,28,29,30,31].

The aim of the study was to evaluate the fatty acid profile and nutraceutical properties of lipids in fallow deer (*Dama dama*) meat produced in organic and conventional farming systems. Additionally, an attempt was made to estimate the nutritional contribution of this type of meat in the diet of adults in terms of the levels of total fat, fatty acids, and cholesterol.

## 2. Materials and Methods

### 2.1. Meat Samples, Animals, and Treatments

Given the special culinary preferences of Polish consumers for two parts of cervid meat, e.g., the loin and leg [32,33], the research material consisted of longissimus lumborum (LL) and semimembranosus (SM) muscles dissected from 12 carcasses of fallow deer reared on an organic farm and 12 carcasses of fallow deer kept in a conventional farming system. In both groups of animals, there were six does and six bucks with an equal age ratio of ca. 18 and 30 months. The farms were located in the area of the Beskid Niski Mts., Podkarpackie Province. The organic farm followed the requirements of Regulation (EU) 2018/848 of the European Parliament and Council of 30 May 2018 [34] and the Act on organic farming (Journal of Laws 2009, No. 116, item 975) [35]. The organic farming certificate covered the grazing area, animals, fodder, and all other rearing procedures. The staple food for the animals was provided by a natural grazing ground with a density of 0.42 LU (large livestock units)/ha. The 191 plant species identified in the pasture [36] represented the following floristic groups: grasses—10.99%, legumes—22.51%, sedges—2.09%, rushes—2.62%, dicotyledonous herbs—44.50%, deciduous trees—6.28%, coniferous trees—1.05%, blackberries—1.05%, shrubs—4.71%, shrublets—2.62%, ferns—0.53%, and horsetails—1.05%. In winter, the animals received hay, straw, cereal grains, and carrots ad libitum.

The animals from the conventional farming system were reared at a density of 0.67 LU/ha following the DEFRA [37] and FEDFA [38] recommendations. The pasture comprised 72 plant species [36] from the following floristic groups: grasses—22.22%, legumes—11.11%, sedges—1.39%, herbaceous dicotyledons—43.05%, deciduous trees—5.56%, coniferous trees—2.78%, blackberries—1.39%, shrubs—6.94%, shrublets—4.17%, and horsetails—1.39%. In winter, the animals received hay, haylage, straw, and fodder beets ad libitum.

The animals were slaughtered by shooting from October to December upon the consent and supervision of veterinary services. The post-slaughter treatments, e.g., evisceration, skinning, and veterinary inspection of carcasses, were carried out after transporting the carcasses in a refrigeration truck to an authorized processing plant. During the dissection of cooled carcasses (cooling for 48 h at a temperature of 4 °C), three equal-sized samples of LL and SM were collected and an approx. 500-g pooled sample placed in polyethylene bags was transported in an isothermal container to the laboratory. The meat was stored at −200 C until laboratory analyses.

### 2.2. Chemical Analysis

Fat was extracted with the method proposed by Folch [39]. Approximately 5 g of meat were homogenized with 5 mL of methanol. The samples were extracted using an automated Soxhlet extractor (Soxtec Avanti, Tecator). The extracted lipids were converted into fatty acid methyl esters (FAME). Fatty acids were saponified with 0.5 N KOH in methanol at 80 °C and then esterified with boron trifluoride/methanol in accordance with the PN-ISO 1444:2000 standard [40]. Separation and quantification of the fatty acid methyl esters was carried out using a gas chromatograph (Varian 450-GC with an FID detector) equipped with a flame ionization detector and fitted with a Select™ Biodiesel for FAME capillary column (30 m × 0.32 mm internal diameter, and 0.52 µm film thickness, Shinwa Inc.) A split/splitless injection system (split ratio of 1:50) and helium as a carrier gas at a flow rate of 1.5 mL/min were used. The injection port and the detector were maintained at 250 and 270 °C, respectively. The column oven temperature was programmed at 100 °C, and finally held at 240 °C for 20 min. The identification of individual FAMEs was based on a standard mixture of 37 Component FAME Mix-CRM47885, St. Louis, MO, USA. The Galaxie ™ Chromatography Data System software was used to convert the results. All samples were analyzed in triplicate. The results were expressed as g/100 g of total identified fatty acids.

The cholesterol content in the muscles was determined with the SOP M.023a method (2011) [41]. The analysis was performed with a gas chromatograph (GC—2010 Shimadzu) equipped with an on-column capillary injector and a flame ionization detector. A capillary column (Zebron ZB-5, L = 30 mm, I.D. = 0.25 mm; df. = 0.5 μm) and a ramped oven temperature were used (increased to 150 °C from 100 °C at 30 °C/min, then increased to 360 °C at 15 °C/min). The cholesterol content was expressed as mg/100 g of fresh meat.

Using the content of individual fatty acids (FA), the following parameters were calculated: SFA—saturated fatty acids, UFA—unsaturated fatty acids, MUFA—monounsaturated fatty acids, PUFA—polyunsaturated fatty acid, PUFA n-6—polyunsaturated fatty acid n-6, PUFA n-3—polyunsaturated fatty acid n-3, OFA—hypercholesterolemic fatty acids = (C12:0 + C14:0 + C16:0), and SFA/UFA; MUFA/SFA; PUFA/SFA; PUFA n-6/PUFA n-3.

The assessment of the nutritional quality of lipids was based on calculation of the following parameters: DFA—desirable fatty acids = (MUFA + PUFA + C18:0), AI—atherogenic index = [C12:0 + 4×C14:0 + C16:0]/[MUFA + PUFA] [42], TI—thrombogenic index = [C14:0 + C16:0 + C18:0]/[0.5×MUFA + 0.5×n-6 + 3×n-3 + n-3/n-6] [42], h/H—ratio of hypo- and hypercholesterolemic fatty acids = [C18:1c9 + C18:2n-6 + C18:3n-3 + C20:3n-6 + C20:4n-6 + C20:5n-3 + C22:5n3]/[C12:0 + C14:0 + C16:0] [7], NV—nutritional value = [C12:0 + C14:0 + C16:0]/[C18:1c9 + C18:2n-6] [43], DHA + EPA—sum of docosahexaenoic acid and eicosapentaenoic acids, HPI—health-promoting index = UFA/[C12:0 +(4 × C14:0) + C16:0] [44], CI—cholesterol index = 1.01 (g of SFA 100 g^−1^ of fresh matter—0.5 × g of PUFA 100 g−1 of fresh matter) + (0.06 × mg of cholesterol 100 g^−1^ of fresh matter) [45], and CSI—cholesterol-saturated fat index = (1.01 × g of SFA 100 g^−1^ of fresh matter) + (0.05 × mg of cholesterol 100 g−1 fresh matter) [46]. The activities of Δ9-desaturase C16 = [C16:1/(16:0 + C16:1)] × 100, Δ9-desaturase C18 [C18:1n9c/(18:0 + C18:1n9c)] × 100, and elongase = [C18:0 + C18:1n9c]/[C16:0 + C16:1+ C18:0 + C18:1n9c] × 100 [29] were calculated as well.

The nutritional contribution of 100 g of fallow deer meat in the diet for adults was estimated by comparison of the total fat and fatty acid content to the recommendations of FAO (2010) [47] and EU (2011) [48]. The cholesterol level was compared with to the recommendations of WHO/FAO (2003) [49].

### 2.3. Statistical Analysis

The numerical data were analyzed with methods of descriptive statistics and statistical hypothesis testing. The arithmetic mean ($\bar{x}$) and standard error (SE) were calculated. The statistical hypothesis testing was preceded by the examination of the normality of selected empirical distributions. The χ^2^ chi-squared test showed that the empirical distributions were consistent with the normal distribution; hence, the hypotheses were verified with the one-way analysis of variance (ANOVA) and the F-test (Fisher–Snedecor). Differences were considered significant at *p* ≤ 0.05. The data were analyzed using Statistica software (v. 13.3, TIBCO Software Inc., Palo Alto, CA, USA).

## 3. Results

**Figure 1:**
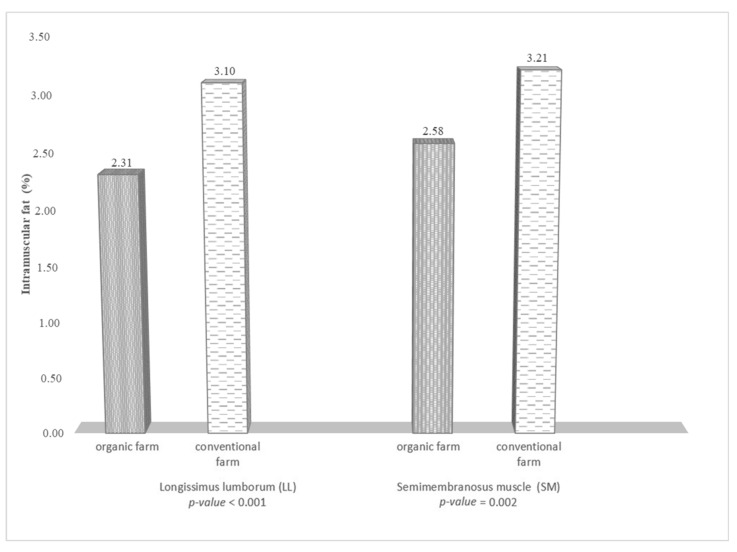
Intramuscular fat content (%) in the meat of fallow deer from organic and conventional farms.

**Figure 2:**
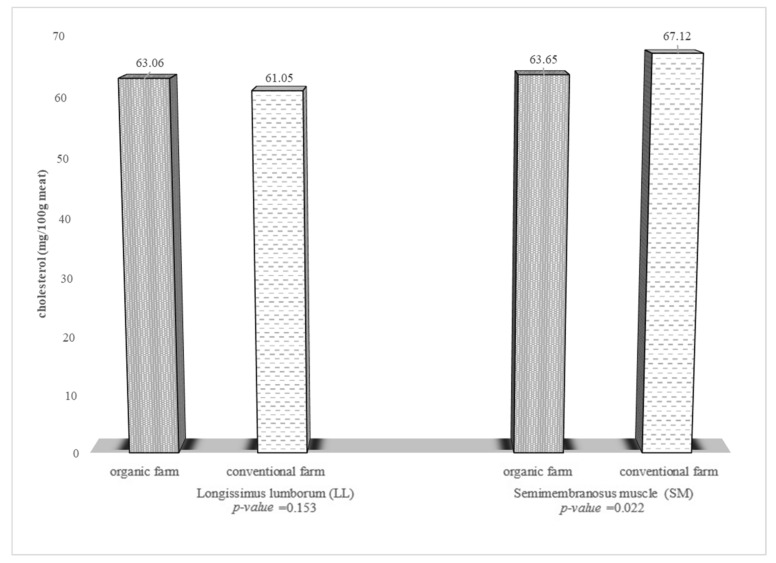
Cholesterol content (mg/100 g meat) in the meat of fallow deer from organic and conventional farms.

Both muscles (LL, SM) of the fallow deer from the organic farm had significantly lower content of intramuscular fat (Figure 1). It was shown that the farming system had a significant impact on the cholesterol content only in the SM muscle (Figure 2). The cholesterol content in the SM muscle of the fallow deer from the organic farm was 3.47 mg/g lower (*p* ≤ 0.022) than in the muscle of the conventionally farmed fallow deer. The cholesterol content in the LL muscle was 2.01 mg/g lower in the fallow deer from the conventional farm (Figure 2).

Table 1 shows the analyzed fatty acids. In comparison with the muscle samples from the conventional farm, the LL muscles of the organically reared fallow deer had a lower concentration of C12:0, C15:0, C18:0, and C21:0 and a higher level of C24:0 (*p* ≤ 0.0001). In turn, significantly higher levels of C12:0, C15:0, C17:0, and C18:0 and lower contents of C21:0 and C24:0 were determined in the SM of the conventionally farmed fallow deer, compared to the SM muscles from the animals reared on the organic farm. The LL muscles of the conventionally farmed fallow deer were characterized by higher concentrations of C15:1, C20:1, and C24:1n-9 than those in the muscles of the other animals. In turn, the SM muscle of the organically farmed animals had lower (*p* ≤ 0.0001) concentrations of C14:1, C15:1, and C20:1 and a higher level of C16:1, compared to the meat produced in the organic farming system. Increased levels of C18:2n-6t, C18:2c9t11, C18:3n-6, C18:3n-3, and C22:6n-3 (*p* ≤ 0.0001) as well as C20:5n-3 (*p* ≤ 0.005) were found in the LL muscle of the fallow deer from the organic farm, whereas the content of C20:3n-6 (*p* ≤ 0.0001) was higher in the LL muscle of the conventionally farmed animals. The organic SM muscle had significantly higher levels of C18:2n-6c, C18:2n-6t, C18:2c9t11, C18:3n-3, C20:3n-6, and C22:6n-3.

In both muscles, the SFA sum was higher in the meat of the conventionally farmed fallow deer, but the differences (*p* ≤ 0.011) were significant only in the SM muscle. The higher SFA sum was reflected in the higher SFA/UFA value (Table 2). The LL and SM muscles of the organically reared fallow deer had higher total PUFA content, including n-6 PUFAs and n-3 PUFAs. Additionally, the organic SM had higher (*p* ≤ 0.011) UFA content than the muscle from the conventional farming system. A higher value of the PUFA/SFA ratio and a lower n-6 PUFA/n-3 PUFA ratio were recorded in the LL and SM muscles of the organically farmed fallow deer. In terms of the nutraceutical properties, the LL and SM muscles of the fallow deer from the organic farm had a higher TI value and higher EPA + DHA content. The cholesterol-saturated fat index (CSI) was significantly higher (*p* ≤ 0.008) in the MS muscle from the conventional system. Significantly higher activity of Δ9-desaturase C16 and lower activity of elongase were determined in both types of muscle of the organically farmed animals (Table 2).

Table 3 shows the FAO (Food and Agriculture Organization) recommended levels of fat and FAs as an energy source in a diet for adults [47] and the relevant EU recommendations for the dietary fat, FA (Fatty acid), and cholesterol intake [48]. In a 2000-kcal daily diet, the consumption of 100 g of fresh fallow meat from the organic farm covered the largest percentage of the EPA + DHA demand (over 28% in SM and 29% in LL). The recommended daily intake of other components was realized as follows: cholesterol (over 21%), n-3 PUFAs (mean 9.15% in SM and 8.81% in LL), fat (mean 4.58% in SM and 4.11% in LL), SFAs (3.81% in SM and 3.49% in LL), and n-6 PUFAs (mean 2.85% of SM and 2.40% of LL). Consumption of the conventionally farmed fallow deer meat was found to cover a slightly higher percentage of fat demand (mean 5.70% in SM and 5.51% in LL) and SFA demand (5.14% and 5.02%).

## 4. Discussion

The samples of muscles were taken during the production process, where the animals were slaughtered to meet the economic needs of the farm owners. The fallow deer were culled in the autumn period in agreement with hunting regulations. The animals were in the age range reported in the literature [51,52]. Fallow deer are usually slaughtered between the 16th and 24th months, due to the highest body weight gains, the most effective feed conversion, low subcutaneous fat cover, and the highest meat quality. In turn, Volpelli et al. [52] highlighted the economic benefits of the extension of fallow deer breeding from 18 to 30 months due to higher dressing proportions, higher amounts of first quality cuts, and better carcass conformation. 

An important indicator of the quality of meat is its fat content. Due to its various functions, fat has an impact on human health; therefore, both excessive levels of total fat in the diet and an imbalance in the fat composition are associated with various diseases. The present study showed that the farming system had an impact on the value of this parameter. Lower fat content was determined in the organic meat, as in the study on beef conducted by Ribas-Augusti et al. [1]. The differences in the content of this component in muscles of wild and farmed deer in Lithuania were reported by Razmaitė et al. [53]. In a study conducted by Daszkiewicz et al. [54], meat from farmed fallow deer had lower intramuscular fat content than meat from wild fallow deer (0.24% vs. 0.50%). Noteworthy, the fat content in the fallow deer meat from the organic and conventional farms analyzed in the present study (Figure 1) was at the optimal level (2–3%) [28]. This is extremely important given the well-documented role of intramuscular fat in the sensory properties of meat [55]. An increase in the intramuscular fat content to the optimal level improves the intensity of meat flavor, juiciness, and tenderness [54]. Joo et al. [56] show a positive correlation between the type IIB fiber and IMF content in Hanwoo steer cattle. The higher fat content determined in the MS muscle compared to the LL muscle (Table 1) may be related to the higher amount of type IIB fibers.

Although there have been no upper limits on cholesterol intake since 2015 (previously <300 mg per day), the dietary guidelines for Americans still recommend the lowest cholesterol intake possible [44]. Thus, for recommended health reasons, the LL was characterized by lower cholesterol content. SM from the conventional vs. organic rearing system had higher cholesterol content. Similarly, Ribas-Augusti et al. [1] reported higher cholesterol content in conventionally farmed beef than in organic beef. Since there are no similar data, comparison with other results of studies of these two cervid production systems is difficult. The higher muscle cholesterol content may be a result of the differences in the diet and the slightly higher total fat content in the meat of the conventionally farmed fallow deer. Chung et al. [57] reported a strong relationship between cholesterol levels and marbling scores. It was found in the present study that the muscles of the analyzed fallow deer generally had lower cholesterol content than the muscles of wild-living deer examined by Polak et al. [30] in the Republic of Slovenia. A higher level of cholesterol was determined in chicken meat (89–129 mg100 g^−1^) [31] and *longissimus dorsi* (LD) and *semitendinosus* (ST) muscle of lambs (99.4–223.28 mg100 g^−1^ and 68.7– 166.2 mg100 g^−1^, respectively) [58].

The biological value of fat is determined primarily by the amount and type of FA contained therein. The fatty acid composition is essential, as it may influence the development of vascular and coronary diseases in humans [59]. As shown by Simopoulos [60], SFAs have been identified as a risk factor for human health. No significant differences in the SFA content were noted in the LL samples from the fallow deer reared in the two farming systems analyzed in the present study. The higher SFA level determined in the SM muscle of the conventionally farmed fallow deer was mainly a consequence of the higher contents of C18:0, C15:0, C17:0, and C12:0. In turn, Revilla et al. [61] reported a higher concentration of SFA in the meat of cattle reared in the organic system compared with the conventional farming system. C16:0 and C18:0 acids are the dominant SFAs in red meat [23,24,52], which was demonstrated in the present study as well. No effect of stearic acid C18:0 on total cholesterol levels has been reported. This is most likely related to its desaturation to oleic acid in the liver [28,54]. However, the thrombogenic properties of C18:0 have been demonstrated [62]. Myristic (C14: 0) and lauric (C12: 0) acids, which were detected in substantially smaller amounts (Table 1), and palmitic acid (C16:0) probably exert atherogenic effects. They inhibit the expression of the LDL (low-density lipoprotein) receptor gene, thus increasing the synthesis of LDL cholesterol and the level of total cholesterol [63]. However, the potential of C14:0 to raise total serum cholesterol is fourfold or even sixfold higher than that of C16:0 [23]. A meta-analysis of results from several studies on the effect of dietary fatty acids has demonstrated that lauric acid increases the level of high-density lipoprotein (HDL) as well [63]. An overall effect of C12:0 is the reduction of the TC-to-HDL ratio, which is associated with desirable cardiovascular outcomes. However, a meta-analysis of prospective epidemiological studies [64] has provided no compelling evidence for the correlation of dietary saturated fat with an increased risk of coronary artery disease (CHD) or cardiovascular disease (CVD). 

Stearic acid contained in meat plays a significant role in meat tenderness and juiciness. As reported by Wood et al. [55,65], there is a positive correlation of meat flavor with the content of saturated and monounsaturated fatty acids and a negative correlation with the level of unsaturated fatty acids. Similar SFA levels to the values reported in the present study were shown by Daszkiewicz et al. [54] in a wild population of fallow deer from northeastern Poland and by Bures et al. [66] for LL of fallow deer from the Czech Republic. A higher level was reported by Daszkiewicz et al. [54] in muscles from a farm-raised population and Ivanović et al. [24] in LT muscles of fallow deer from Serbia.

No differences in the content of monounsaturated fatty acids (MUFAs) were observed between the muscles from the conventional and organic farming systems. However, the C16:1 concentration was higher in the LL and SM muscles from the organically than conventionally farmed animals, as in the study on beef conducted by Ribas-Augusti et al. [1]. The high content of this FA may be related to the increased activity of stearoyl-CoA desaturase Δ9 (Δ9-desaturase C16) (Table 2). It is one of the most important endoplasmic reticulum (ER)-associated enzymes catalyzing the generation of monounsaturated fatty acids (MUFAs; C16:1 n-7) from saturated fatty acids (SFAs; C16: 0) synthesized *de novo* or supplied with food [67]. This is an important issue since increasing attention is being paid to the possibility of using stearoyl-CoA desaturase in the treatment of circulatory diseases and cancers [68,69].

Essential PUFAs are not synthesized in the human organism, and their deficiency in the diet causes metabolic and health disorders. PUFAs are classified into four families: n-3, n-6, n-9, and n-7. In terms of nutrition, α-linolenic acid (ALA; 18:3 n-3) and linoleic acid (LA; 18:2 n-6), which are precursors of long-chain polyunsaturated fatty acids (LCPUFA) are the most important PUFAs. The process of biochemical transformations of ALA leads to formation of EPA (20:5n-3) and DHA (22:6n-3), whereas LA is converted into arachidonic acid (ARA; 20:4n-6). The synthesis is possible due to the presence of appropriate enzymes [70]. LA and ALA compete for the same desaturase and elongase enzymes involved in the synthesis of LCPUFA [44].

The present study showed that the muscles from the organically farmed animals had a higher content of n-3 PUFAs (ALA and DHA) and CLA and C18:2n-6t compared with the muscles of fallow deer from the conventional system. The organic LL samples had a higher concentration of EPA and GLA, and the SM muscles contained higher amounts of LA. High PUFA content in meat is desirable due to their nutritional value and health-enhancing properties. A higher level of n-3 PUFAs and CLA was also noted by Revilla et al. [61] and Turner et al. [71] in organic beef as well as Kamihiro et al. [27], who observed differences only in the content of n-3 PUFAs. PUFAs act as carriers of fat-soluble vitamins (A, D, E, and K) and play a key role in the immune response in humans and animals [72]. They are involved in vital metabolic processes such as brain development [73], endocytosis and exocytosis, and cellular signal transduction [74]. ALA is involved in prevention of cardiovascular diseases, and LA has 2–3-fold higher efficiency in lowering the LDL-C level than oleic acid [75]. 

EPA and DHA exert a hypolipidemic effect by reducing the concentration of triglycerides (TG) in blood plasma via inhibition of their resynthesis in the intestinal wall and liver and activate anti-inflammatory, anticoagulant, and other anti-atherosclerotic mechanisms [44,70,76]. EPA and DHA play a beneficial role in many human diseases, including autoimmune diseases, diabetes, cancer, and Alzheimer’s disease (AD) [76,77]. EPA exerts an effect mainly on the cardiovascular system through the synthesis of eicosanoids. At the same time, DHA is an important structural component of nervous cell membranes, especially in the brain cortex and retina [44]. Moreover, a beneficial role of DHA in counteracting depression and stress has been indicated [70,73]. It is also suggested that omega-3 acids reduce the severity of viral and bacterial inflammatory processes [70]. These data suggest that organic fallow deer meat characterized by the significantly higher level of DHA + EPA (Table 2) should be included in the human diet more frequently.

Recently, considerable attention has been paid to CLA due to its proven bioactivity in the prevention of obesity, cancer, diabetes, atherosclerosis, and osteoporosis [44,78,79]. Conjugated linoleic acid is synthesized by the bacterium *Butyrivibrio fibrisolvens* in the rumen of ruminants through incomplete hydrogenation of linoleic acid to stearic acid [80]. The present study shows that the LL and SM muscles of fallow deer from the organic farming system were characterized by significantly higher CLA levels, which may be associated with the great floristic diversity of the pasture and the consumption of a higher number of grass, legume, and herb species. Higher CLA concentrations in the meat of grazing animals than those receiving feed concentrates were observed in other studies as well [7,61]. As reported by Budimir et al. [26], consumption of grass and grazing significantly increased the concentration of CLA, n-3 PUFAs, and MUFA in lamb meat.

The PUFA/SFA, n-6 PUFA/n-3 PUFA, and h/H ratios are commonly used for the assessment of the IMF nutritional value and consumer health. All dietary PUFAs are believed to lower the levels of low-density lipoprotein cholesterol (LDL-C) and total cholesterol in serum, while all SFAs contribute to the elevation of serum cholesterol levels. Thus, higher ratios are correlated with more positive effects [44]. According to the relevant nutritional recommendations, the PUFA/SFA ratio in the human diet should be 0.4 or higher [55]. Some authors [81] suggest that this ratio should be in the range from ≥ 0.45 to 1.0. In the present study, these criteria were fulfilled only by the meat from the organically farmed fallow deer. Foods in the human diet with a PUFA/SFA ratio below 0.45 are regarded as undesirable due to their potential effect of increasing blood cholesterol levels [82]. In the present study, the PUFA/SFA ratio in the meat of the conventionally reared fallow deer was similar to the value reported by Ivanović et al. [24] for the musculus longissimus thoracis (LT) of fallow deer from Serbia. In turn, the ratio in the muscles of the organically farmed animals was higher than the value in meat from farmed and wild fallow deer (0.27) calculated by Daszkiewicz [52] and in beef and lamb muscles (0.11–0.37) [44]. As suggested by Simopoulos [60], a balanced n-6/n-3 ratio of 1–2/1 is one of the most important dietary factors in the prevention of obesity, whereas dieticians claim that the desired n-6/n-3 ratio should be 5. As reported by Harris [83], there are many indications that the ratio of dietary n-3 and n-6 acids is irrelevant, and its role in prevention of many diseases is unreliable. Based on the available literature [47,83], it can be concluded that achievement of an appropriate threshold of consumption of n-3 and n-6 fatty acids has fundamental importance. The diet of Western societies has been shown to be deficient in n-3 acids [60,73]. It is now known that n-3 fatty acids are highly important for the proper growth and development of the human organism. The present study showed a higher concentration of n-6 PUFAs and n-3 PUFAs and a lower n-6 PUFA/n-3 PUFA ratio in the organic meat; nevertheless, the concentration of these FAs was high in the meat from both production systems, which undoubtedly proves that fallow deer meat is a good source of n-6 PUFAs and n-3 PUFAs. Therefore, it can be recommended for the prevention and treatment of such diseases as hypertension, diabetes, arthritis, inflammatory diseases, coronary heart disease, and cancers [70,80]. Similarly, lower n-6/n-3 values in organic than conventional beef were reported by Revilla et al. [61] and Turner et al. [71].

Ulbricht and Southgate [42] have found that AI and TI indices are better indicators of atherogenicity and thrombogenicity than the PUFA/SFA ratio. In general, their lower value is more beneficial for health. This is associated with the fact that not all SFAs are hypercholesterolemic, and MUFAs exert a protective effect, likewise PUFAs. It is assumed that an AI value lower than 0.5 is beneficial for human health [84], although some authors recommend an atherogenicity index for animal lipids in the range from 0.5 to 1.0 [81,85]. In the present study, the values of AI of the organically and conventionally produced meat did not differ significantly. They were consistent with the dietary recommendations [60,73] and agreed with the results reported by Švrčula et al. [85]. This is extremely important from the health point of view, as consumption of products with a lower AI value may contribute to the reduction of the level of total cholesterol and LDL-C in human blood plasma [44].

The TI values calculated for the LL and SM muscles of the organically farmed fallow deer were lower and more favorable than in the conventional farming system. However, they were within the recommended range for a healthy diet (<1.0) in both systems [73]. Noteworthy, the fat in the organic meat appeared to have very low thrombogenicity and atherogenicity indices similar to those of seafood [86]. The TI values recorded in this study were lower than the indices for fallow deer meat (1.42–1.83) from the Czech Republic [85] as well as lamb and heifer meat (1.1–1.34) [44]. The TI value reflects the thrombogenic potential of FAs, indicating a tendency to form clots in blood vessels, and reveals the contribution of various FAs and the relationship between pro-thrombogenic FAs (C12:0, C14:0, and C16:0) and anti-thrombogenic FAs (MUFAs and the n-3 and n-6 families). Therefore, the consumption of meat with a lower TI value is beneficial for CVH [44]. FAs with lower AI and TI values have better nutritional quality, and consumption of such fatty acids may reduce the risk of coronary heart disease (CHD). Nevertheless, no specific AI and TI values have been recommended to date [44].

The hypercholesterolemic and atherosclerotic potential of meat is associated with the content of cholesterol and saturated fats. Hence, the CI and CSI indices depending mainly on the cholesterol content and, to a lesser extent, on the level of fat and SFA concentration, were analyzed in this study. These indices have been proposed as useful elements of evaluation and design in low-fat diets, and offer quick and easy assessments of daily cholesterol intake. Their low value reflects low contents of saturated fat and cholesterol and thus low atherogenicity [46]. The SM muscles from the organically farmed fallow deer were characterized by significantly reduced CI and CSI values, indicating that fallow deer meat has great potential to reduce hyperlipidemia. Therefore, the meat of fallow deer kept in organic systems can be regarded as a functional food due to its content of fatty acids and the reduced risk of cardiovascular and autoimmune diseases [70,77]. The EPA and DHA fatty acids are essential parameters for recognizing the organic meat analyzed in this study as a functional food [76,77]. It was estimated that a 100-g portion of fresh organic fallow deer meat ensured nearly 30% of the recommended daily intake of EPA+DHA for adults; therefore, it can be labeled as meat with high PUFA n-3 content. Venison is currently an exclusive product and, although Poland is one of the leading game producers in Europe, its average annual consumption per capita in our country ranges from 50 g/person (2013) to 138 g/person (2016) [87].

The present investigations of the quantity and quality of lipids in fallow deer meat show that the natural conditions of animal husbandry following the organic farming principles can provide food with a higher health-enhancing value.

## 5. Conclusions

The organic fallow deer meat was characterized by a significantly lower content of intramuscular fat. The fatty acid profile in the organic meat exhibited a particularly high proportion (*p* <0.0001) of CLA (LL—2.29%, SM—2.14%), ALA (LL—4.32%, SM—3.87%), and DHA (LL—2.83%, SM—2.60%). The beneficial effect (*p* < 0.0001) of the organic farming system on the amount of PUFAs, including n-3 PUFAs, was shown to result in a more favorable n-6 PUFA/n-3 PUFA ratio. The significantly higher nutritional quality of organic meat lipids was confirmed by such nutraceutical indicators as TI, ∆9-desaturase C16, elongase, and DHA + EPA for the LL and SM muscles and the CI and CSI indices for the SM muscle. A 100-g portion of fresh organic fallow deer meat was found to provide nearly 30% of the recommended daily intake of EPA + DHA acids in the adult diet. Regardless of the farming system, the longissimus lumborum muscle turned out to be more attractive to consumers in terms of its health-enhancing properties.

## Figures and Tables

**Table 1:** Fatty acid profile (g·100 g^−1^ FA; mg·100 g^−1^ fresh meat) in the meat of fallow deer from organic and conventional farms.

Fatty Acids	Muscle													
	*longissimus lumborum* (LL)							*semimembranosus* (SM)						
	Organic Farm			Conventional Farm				organic Farm			Conventional Farm			
	$\bar{x}$	SE	mg·100 g^−1^	$\bar{x}$	SE	mg·100g^−1^	*p*-Value	$\bar{x}$	SE	mg·100g^−1^	$\bar{x}$	SE	mg·100g^−1^	*p*-Value
C10:0	0.09	0.022	1.45	0.07	0.013	1.66	0.123	0.09	0.026	1.64	0.08	0.013	1.71	0.180
C12:0	0.16	0.020	2.65	0.23	0.082	5.10	0.006	0.16	0.033	2.94	0.23	0.083	5.23	0.019
C13:0	0.19	0.223	3.09	0.18	0.261	3.87	0.967	0.17	0.207	2.96	0.24	0.344	5.31	0.571
C14:0	3.44	1.316	58.38	3.84	1.531	83.02	0.503	3.39	1.534	63.57	3.72	1.045	87.25	0.551
C15:0	0.87	0.393	13.90	1.49	0.492	33.11	0.002	0.38	0.256	7.33	1.74	0.253	40.14	<0.0001
C16:0	23.96	2.424	398.55	22.49	2.376	500.72	0.146	21.66	2.732	399.67	20.46	3.288	467.10	0.341
C17:0	1.10	0.506	17.33	0.86	0.281	18.70	0.154	0.51	0.238	9.59	1.23	0.418	28.99	<0.0001
C18:0	15.52	4.780	254.27	19.07	3.491	472.42	0.050	17.80	2.896	328.77	20.53	3.405	477.84	0.046
C20:0	0.30	0.220	5.12	0.50	0.298	11.51	0.069	0.21	0.067	3.83	0.23	0.103	5.33	0.578
C21:0	0.23	0.119	4.02	0.75	0.110	6.44	<0.0001	0.33	0.106	5.97	0.22	0.073	4.96	0.006
C22:0	0.07	0.029	1.18	0.08	0.013	1.75	0.593	0.13	0.041	2.34	0.22	0.144	5.00	0.055
C24:0	0.18	0.033	2.91	0.09	0.023	1.90	<0.0001	0.17	0.034	3.07	0.10	0.027	2.32	<0.0001
C14:1	2.57	0.232	42.65	2.87	0.839	64.09	0.248	1.63	0.409	29.60	3.29	1.186	76.82	<0.0001
C15:1	1.23	0.386	20.93	2.50	0.816	54.28	<0.0001	1.08	0.896	19.87	3.59	1.277	84.18	<0.0001
C16:1	3.56	0.927	57.83	1.90	1.155	41.53	0.001	3.89	0.942	70.99	2.11	1.138	49.58	<0.0001
C17:1	1.31	0.360	21.19	0.67	0.336	14.61	<0.0001	1.04	0.302	18.94	0.86	0.342	20.22	0.186
C18:1n9c	19.95	4.833	336.86	22.86	3.039	513.16	0.092	23.26	3.239	432.12	22.77	3.227	522.55	0.716
C18:1n9t	0.48	0.250	8.28	0.70	0.282	15.88	0.057	0.75	0.994	13.04	0.62	0.390	14.89	0.683
C20:1	0.23	0.119	4.01	0.75	0.110	16.82	<0.0001	0.16	0.073	2.98	0.64	0.088	14.80	<0.0001
C24:1n9	0.19	0.034	3.13	0.23	0.036	5.17	0.004	0.19	0.050	3.41	0.16	0.041	3.69	0.183
C18:2n6c (LA)	7.64	0.966	128.31	6.99	1.419	156.99	0.208	7.24	1.022	133.61	5.97	1.659	142.21	0.035
C18:2n6t	0.63	0.052	10.54	0.36	0.061	7.95	<0.0001	0.60	0.036	10.99	0.33	0.068	7.88	<0.0001
C18:2c9t11 (CLA)	2.29	0.123	37.97	1.60	0.078	35.86	<0.0001	2.14	0.343	39.22	1.66	0.073	38.50	<0.0001
C20:2n6	0.16	0.031	2.63	nd	nd	nd	-	0.15	0.046	2.74	nd	nd	nd	-
C22:2n6	0.32	0.236	5.02	nd	nd	nd	-	0.16	0.065	2.89	nd	nd	nd	-
C18:3n6 (GLA)	1.31	0.157	21.58	0.36	0.196	8.21	<0.0001	1.43	0.461	25.99	1.38	0.235	32.39	0.745
C18:3n3 (ALA)	4.32	0.691	71.93	2.15	0.694	48.92	<0.0001	3.87	0.742	70.81	1.96	0.737	46.63	<0.0001
C20:3n6	0.91	0.237	15.49	1.65	0.263	39.94	<0.0001	0.85	0.170	15.64	0.45	0.221	10.43	<0.0001
C20:3n3	0.64	0.142	10.60	0.73	0.114	16.38	0.079	0.91	0.547	16.35	0.81	0.102	18.69	0.544
C20:4n6 (AA)	1.68	0.146	28.04	1.71	0.065	38.21	0.545	1.75	0.077	32.31	1.74	0.068	40.36	0.781
C20:5n3 (EPA)	1.56	0.464	26.17	1.14	0.037	25.34	0.005	1.23	0.129	22.88	1.19	0.028	25.50	0.240
C22:6n3 (DHA)	2.83	0.093	47.22	1.63	0.072	36.42	<0.0001	2.60	0.083	47.01	1.50	0.029	34.82	<0.0001

nd = not detected.

**Table 2:** Sums of FA groups (g·100 g^−1^ FA), fatty acid ratios, and nutraceutical indices of lipids in the meat of fallow deer from organic and conventional farms.

Specification	Muscle									
	*Longissimus lumborum* (LL)					*Semimembranosus* (SM)				
	Organic Farm		Conventional Farm		*p*-Value	Organic Farm		Conventional Farm		*p*-Value
	$\bar{x}$	SE	$\bar{x}$	SE		$\bar{x}$	SE	$\bar{x}$	SE	
**Sums of FA groups**										
SFA	46.12	5.742	49.19	4.790	0.169	44.98	3.673	48.96	3.332	0.011
UFA	53.88	5.742	50.81	4.791	0.169	55.02	3.673	51.04	3.332	0.011
MUFA	29.62	5.096	32.49	3.390	0.119	32.10	3.610	34.04	3.294	0.183
PUFA	24.26	1.822	18.33	2.256	<0.0001	22.92	2.350	17.00	2.502	<0.0001
PUFA n-6	12.59	1.457	11.05	1.624	0.023	12.18	1264	9.88	1.991	0.003
PUFA n-3	9.34	0.565	5.65	0.744	<0.0001	8.60	1.180	5.46	0.740	<0.0001
OFA	27.56	1.631	26.56	2.988	0.318	25.21	3.090	24.40	3.421	0.548
**FA Ratios**										
SFA/UFA	0.88	0.199	0.99	0.218	0.204	0.83	0.123	0.97	0.139	0.014
MUFA/SFA	0.66	0.208	0.67	0.115	0.938	0.72	0.129	0.70	0.099	0.647
PUFA/SFA	0.54	0.094	0.38	0.073	<0.0001	0.51	0.081	0.35	0.064	<0.0001
PUFA n-6/PUFA n-3	1.35	0.123	1.96	0.176	<0.0001	1.42	0.124	1.81	0.269	<0.0001
**Nutraceutical indices**										
DFA	69.40	1.921	69.88	3.261	0.666	72.81	3.265	71.57	3.284	0.361
AI	0.54	0.090	0.54	0.132	0.979	0.47	0.116	0.50	0.090	0.561
TI	0.73	0.153	0.92	0.209	0.022	0.72	0.112	0.90	0.118	<0.0001
h/H	2.53	0.227	2.68	0.426	0.310	2.94	0.470	3.00	0.530	0.771
∆9-desaturase C16	13.01	3.692	7.59	3.978	0.002	15.24	3.527	9.24	4.082	<0.0001
∆9-desaturase C18	56.27	13.044	54.61	6.913	0.701	56.68	4.607	52.62	7.262	0.116
Elongase	56.31	3.369	63.25	3.638	<0.0001	61.52	5.237	65.88	3.909	0.030
NV	1.04	0.224	0.91	0.221	0.186	0.84	0.180	0.86	0.142	0.835
DHA + EPA	4.38	0.477	2.77	0.076	<0.0001	3.83	0.137	2.69	0.027	<0.0001
HPI	1.37	0.190	1.34	0.304	0.747	1.55	0.354	1.42	0.226	0.277
CI	4.35	0.221	4.56	0.218	0.420	4.44	0.224	4.96	0.270	<0.0001
CSI	3.92	0.194	4.15	0.221	0.067	4.02	0.207	4.49	0.276	<0.0001

**Table 3:** Contribution of a 100-g portion of fallow deer meat in the diet of adults in terms of total fat, fatty acids, and cholesterol.

Specification	Percent (%) of Energy Requirements Recommended by FAO ^a^	g/day (in a 2000-Kcal Diet) ^b^	Mean Content (g/100g of Fresh Meat)^c^				Percent (%) of Contribution to a 2000-Kcal Diet			
			*longissimus lumborum* (LL)		*semimembranosus* (SM)		*longissimus lumborum* (LL)		*semimembranosus* (SM)	
			organic Farm	conventional Farm	Organic Farm	Conventional Farm	Organic Farm	Conventional Farm	Organic Farm	Conventional Farm
Total fat	20.0–35.0	44.0–78.0	2.31	3.10	2.58	3.21	2.96–5.25	3.97–7.04	3.30–5.86	4.11–7.29
∑ SFA	<10.0	<22.0	0.768	1.100	0.837	1.133	≥3.49	≥5.02	≥3.81	≥5.14
∑ MUFA	15.0–20.0	33.0–44.0	0.493	0.726	0.597	0.788	1.12–1.49	1.65–2.20	1.36–1.81	1.79–2.39
∑ PUFA	6.0–11.0	13.0–24.0	0.404	0.410	0.426	0.393	1.68–3.11	1.71–3.15	1.77–3.27	1.64–3.02
∑ PUFA n-6	2.5–9.0	5.6–20.0	0.210	0.247	0.226	0.228	1.05–3.75	1.23–4.42	1.13–4.03	1.14–4.07
∑ PUFA n-3	0.5–2.0	1.1–4.4	0.155	0.126	0.161	0.126	3.52–14.09	2.86–11.45	3.66–14.64	2.86–11.54
EPA + DHA ^d^	250 mg	0.250	0.073	0.062	0.071	0.062	29.23	24.81	28.41	24.81
Cholesterol ^e^	<300 mg	<0.300	0.063	0.061	0.063	0.067	≥21.02	≥20.35	≥21.22	≥22.37

^a^ FAO (2010); ^b^ European Union (EU) (2010); ^c^ FA content expressed in g/100 g of muscle tissue calculated from total intramuscular fat (IMF) using a conversion factor of (*F*_CON_) 0.721 [50]; ^d^ The upper level of EPA + DHA consumption should not exceed 2 g/day; ^e^ WHO/FAO (2003).

## Data Availability

The data presented in this study are available in the article.

## References

[1] Ribas-Agustí A., Díaz I., Sárraga C., García-Regueiro J.A., Castellari M. (2019). Nutritional properties of organic and conventional beef meat at retail. J. Sci. Food Agric..

[2] Rizzo G., Borrello M., Dara Guccione G., Schifani G., Cembalo L. (2020). Organic Food Consumption: The Relevance of the health attribute. Sustainability.

[3] Sahota A., Willer H., Lernoud J. (2018). The world of Organic Agriculture. Statistics and Emerging Trends 2018.

[4] Tandon A., Jabeen F., Talwar S., Sakashita M., Dhir A. (2021). Facilitators and inhibitors of organic food buying behavior. Food Qual. Prefer..

[5] Hurtado-Barroso S., Tresserra-Rimbau A., Vallverdú-Queralt A., Lamuela-Raventós R.M. (2019). Organic food and the impact on human health. Crit. Rev. Food Sci. Nutr..

[6] Anisimova T., Mavondo F., Weiss J. (2017). Controlled and uncontrolled communication stimuli and organic food purchases: The mediating role of perceived communication clarity, perceived health benefits, and trust. J. Mark. Commun..

[7] Santos-Silva J., Bessa R.J., Santos-Silva F. (2002). Effect of genotype, feeding system and slaughter weight on the quality of light lambs. II. Fatty acid composition of meat. Livest. Product. Sci..

[8] Barański M., Rempelos L., Iversen P.O., Leifert C. (2017). Effects of organic food consumption on human health; the jury is still out!. Food Nutr. Res..

[9] Ditlevsen K., Sandøe P., Lassen J. (2019). Healthy food is nutritious, but organic food is healthy because it is pure: The negotiation of healthy food choices by Danish consumers of organic food. Food Qual. Prefer..

[10] Kilar M., Kilar J., Ruda M., Tarko T. (2016). Rolnictwo ekologiczne jako źródło żywności funkcjonalnej. Innowacyjne Rozwiązania w Technologii Żywności i Żywieniu Człowieka.

[11] Kesse-Guyot E., Péneau S., Méjean C., De Edelenyi F.S., Galan P., Hercberg S., Lairon D. (2013). Profiles of organic food consumers in a large sample of French adults: Results from the Nutrinet-Santé cohort study. PLoS ONE.

[12] Rembiałkowska E., Wiśniewska K. (2010). Jakość mięsa z produkcji ekologicznej. Med. Wet..

[13] Gadomska J., Sadowski S., Buczkowska M. (2014). Ekologiczna żywność jako czynnik sprzyjający zdrowiu. Probl. Hig. I Epidemiol..

[14] Vargas-Ramella M., Munekata P.E.S., Gagaoua M., Franco G., Campagnol P.C.B., Pateiro M., Da Silva Barretto A.C., Domínguez R., Lorenzo J. (2020). Inclusion of healthy oils for improving the nutritional characteristics of dry-fermented deer sausage. Foods.

[15] Żochowska-Kujawska J. (2017). Mięso Zwierząt Łownych Jako Potencjalne Źródło Surowca do Produkcji Surowych Wędzonek Dojrzewających.

[16] Kudrnáčová E., Bartoň L., Bureš D., Hoffman L.C. (2018). Carcass and meat characteristics from farm-raised and wild fallow deer (*Dama dama*) and red deer (*Cervus elaphus*): A review. Meat Sci..

[17] Mexia I.A., Quaresma M.A.G., Coimbra M.C.P., Dos Santos F.A., Alves S.P.A., Bessab R.J.B., Antunes I.C. (2020). The influence of habitat and sex on feral fallow deer meat lipid fraction. J. Sci. Food Agric..

[18] Cawthorn D.M., Fitzhenry L.B., Kotrba R., Bureš D., Hoffman L.C. (2020). Chemical composition of wild fallow deer (*Dama dama*) meat from South Africa: A preliminary evaluation. Foods.

[19] Kilar J. (2018). Dziczyzna w jadłospisie człowieka–walory technologiczne, kulinarne i prozdrowotne. Nowoczesna Produkcja Świń i Stojące Przed nią Wyzwania.

[20] Soriano A., Sánchez-García C. (2021). Nutritional Composition of Game Meat from Wild Species Harvested in Europe. Intech Open.

[21] Kilar J., Ruda M., Kilar M., Grych K., Słupski J., Tarko T., Drożdż I. (2018). Dziczyzna. Szanse i bariery zwiększenia spożycia. Surowce Pochodzenia Zwierzęcego Jako Źródło Składników Bioaktywnych.

[22] Stanisz M., Skorupski M., Ślósarz P., Bykowska-Maciejewska M., Składanowska-Baryza J., Stańczak Ł., Krokowska-Paluszak M., Ludwiczak A. (2019). The seasonal variation in the quality of venison from wild fallow deer (*Dama dama*)—A pilot study. Meat Sci..

[23] Daszkiewicz T., Mesinger D. (2018). Fatty acid profile of meat (*Longissimus lumborum*) from female roe deer (*Capreolus capreolus* L.) and red deer (*Cervus elaphus* L.). Int. J. Food Prop..

[24] Ivanović S., Pisinov B., Pavlović M., Pavlović I. (2020). Quality of meat from female fallow deer (*Dama dama*) and roe deer (*Capreolus capreolus*) hunted in Serbia. Ann. Anim. Sci..

[25] Bykowska M. (2018). Influence of selected factors on meat quality from farm-raised and wild fallow deer (*Dama dama*): A review. Can. J. Anim. Sci..

[26] Budimir K., Mozzon M., Toderi M., D’Ottavio P., Trombetta M.F. (2020). Effect of breed on fatty acid composition of meat and subcutaneous adipose tissue of light lambs. Animals.

[27] Kamihiro S., Stergiadis S., Leifert C., Eyre M.D., Butler G. (2015). Meat quality and health implications of organic and conventional beef production. Meat Sci..

[28] Kasprzyk A., Tyra M., Babicz B. (2015). Fatty acid profile of pork from a local and a commercial breed. Arch. Anim. Breed..

[29] Nogales S., Bressan M.C., Delgado J.V., Telo daGama L., Barba C., Camacho M.E. (2017). Fatty acid profile of feral cattle meat. Ital. J. Anim. Sci..

[30] Polak T., Rajar A., Gaperlin L., Lender B. (2008). Cholesterol concentration and fatty acid profile of red deer (*Cervus elaphus*) meat. Meat Sci..

[31] Rozbicka-Wieczorek A.J., Więsyk E., Brzóska F., Śliwiński B., Kowalczyk J., Czauderna M. (2014). Fatty acid profile and oxidative stress of thigh muscles in chickens fed the ration enriched in lycopene, selenium compounds or fish oil. Ann. Anim. Sci..

[32] Kilar J., Ruda M., Kilar M. (2016). Dziczyzna. Co o Niej Wiedzą i Czy ją Jedzą Mieszkańcy Podkarpacia.

[33] Kwiecińska K., Kosicka-Gębska M., Gębski J. (2016). Ocena preferencji konsumentów związanych z wyborem dziczyzny. Handel Wewnętrzny.

[34] Regulation (EU) 2018/848—Rules on Organic Production and Labelling of Organic Products. EU Rules on Producing and Labelling Organic Products. https://eur-lex.europa.eu/legal-content/EN/LSU/?uri=CELEX%3A32018R0848.

[35] Dz.U. 2009 nr 116 poz. 975. Ustawa z Dnia 25 Czerwca 2009 r. o Rolnictwie Ekologicznym.

[36] Braun-Blanguet J. (1964). Pflanzensoziologie, Grundzuge der Vegetationskundle.

[37] (2006). DEFRA Code of Recommendations for the Welfare of Farmed Deer. http://www.defra.gov.uk/animalh/welfare/farmed/othersps/deer/pb0055/deercode.htm.

[38] FEDFA Federation of European Deer Farmers Associations. https://www.fedfa.com/.

[39] Folch J., Lees M., Sloane-Stanley G.H. (1957). A simple method for the isolation and purification of total lipids from animal tissues. J. Biol. Chem..

[40] (2000). PN-ISO 1444:2000. Meat and Meat Products. Determination of Fat Content.

[41] SOP (2011). Cholesterol in meat by GC method.

[42] Ulbricht T.L.V., Southgate D.A.T. (1991). Coronary heart disease: Seven dietary factors. Lancet.

[43] Domaradzki P., Żółkiewski P., Litwińczuk A., Florek M., Dmoch M. (2019). Profil i wartość odżywcza kwasów tłuszczowych w wybranych mięśniach szkieletowych buhajków rasy polskiej holsztyńsko-fryzyjskiej. Med. Weter..

[44] Chen J., Liu H. (2020). Nutritional indices for assessing fatty acids: A mini-review. Int. J. Mol. Sci..

[45] Zilversmit D.B. (1979). Cholesterol index of foods. J. Am. Diet. Assoc..

[46] Connor S.L., Gustafson J.R., Artaud-Wild S.M., Favell D.P., Classick-Kohn C.J., Hatcher L.F., Connor W.E. (1986). The cholesterol/saturated-fat index an indication of the hypercholesterolemic and atherogenic potential of food. Lancet.

[47] FAO (2010). Fats and Fatty Acids in Human Nutrition: Report of an Expert Consultation. FAO Food Nutr. Pap..

[48] European Food Safety Authority (2010). Scientific opinion on dietary reference values for fats, including saturated fatty acids, polyunsaturated fatty acids, monounsaturated fatty acids, trans fatty acids, and cholesterol. EFSA J..

[49] WHO/FAO (2003). Diet, nutrition and the prevention of chronic diseases. Report of a Joint WHO/FAO Expert Consultation.

[50] Domaradzki P., Florek M., Skałecki P., Litwińczuk A., Kędzierska-Matysek M., Wolanciuk A., Tajchman K. (2019). Fatty acid composition, cholesterol content and lipid oxidation indices of intramuscular fat from skeletal muscles of beaver (*Castor fiber* L.). Meat Sci..

[51] Janiszewski P., Bogdaszewska Z., Bogdaszewski M., Bogdaszewski P., Ciululko-Dołęga J., Nasiadka P., Steiner Ż. (2014). Rearing and Breeding of Cervids on Farms.

[52] Volpelli L.A., Valusso R., Piasentier E. (2002). Carcass quality in male fallow deer (*Dama dama*): Effects of age and supplementary feeding. Meat Sci..

[53] Razmaitė V., Pileckas V., Šiukščius A., Juškiene V. (2020). Fatty acid composition of meat and edible offal from free-living red deer (*Cervus elaphus*). Foods.

[54] Daszkiewicz T., Hnatyk N., Dąbrowski D., Janiszewski P., Gugołek A., Kubiak D., Śmiecińska K., Winarski R., Koba-Kowalczyk M. (2015). A comparison of the quality of the *Longissimus lumborum* muscle from wild and farm-raised fallow deer (*Dama dama* L.). Small Rumin. Res..

[55] Wood J.D., Enser M., Fisher A.V., Nute G.R., Sheard P.R., Richardson R.I., Hughes S.I., Whittington F.M. (2008). Fat deposition, fatty acid composition and meat quality: A review. Meat Sci..

[56] Joo S.H., Lee K.W., Hwang Y.H., Joo S.T. (2017). Histochemical characteristics in relation to meat quality traits of eight major muscles from Hanwoo steers. Korean J. Food Sci. Anim. Resour..

[57] Chung K.Y., Lunt D.K., Choi C.B., Chae S.H., Rhoades R.D., Adams T.H. (2006). Lipid characteristics of subcutaneous adipose tissue and *M. Longissimus thoracis* of Angus and Wagyu steers fed to US and Japanese endpoints. Meat Sci..

[58] Aksoy Y., Çiçek Ü., Sen U., Sirin E., Ugurlu M., Önenç A., Kuran M., Ulutas Z. (2019). Meat production characteristics of Turkish native breeds: II. meat quality, fatty acid, and cholesterol profile of lambs. Arch. Anim. Breed..

[59] Calder P.C., Deckelbaum R.J. (2003). Fat as a physiological regulator: The news gets better. Curr. Opin. Clin. Nutr. Metab. Care.

[60] Simopoulos A. (2016). An increase in the omega-6/omega-3 fatty acid ratio increases the risk for obesity. Nutrients.

[61] Revilla I., Plaza J., Palacios C. (2021). The effect of grazing level and ageing time on the physicochemical and sensory characteristics of beef meat in organic and conventional production. Animals.

[62] Attia Y.A., Al-Harthi M.A., Korish M.A., Shiboob M.M. (2017). Fatty acid and cholesterol profiles, hypocholesterolemic, atherogenic, and thrombogenic incides of broiler meat in the retail market. Lipids Health Dis..

[63] Dayrit F.D. (2015). The properties of lauric acid and their significance in coconut oil. Review. J. Am. Oil Chem. Soc..

[64] Siri-Tarino P.W., Sun Q., Hu F.B., Krauss R.M. (2010). Meta-analysis of prospective cohort studies evaluating the association of saturated fat with cardiovascular disease. Am. J. Clin. Nutr..

[65] Wood J.D., Richardson R.I., Nute G.R., Fisher A.V., Campo M.M., Kasapidou E., Sheard P.R., Enser M. (2004). Effects of fatty acids on meat quality: A review. Meat Sci..

[66] Bureš D., Bartoň L., Kotrba R., Hakl J. (2015). Quality attributes and composition of meat from red deer (*Cervus elaphus*), fallow deer (*Dama dama*) and Aberdeen Angus and Holstein cattle (*Bos taurus*). J. Sci. Food Agric..

[67] Kucharski M., Kaczor U. (2014). Stearoyl-CoA desaturase—The lipid metabolism regulator. Postepy Hig. Med. Dosw..

[68] Mayneris-Perxachs J., Guerendiain M., Castellote A.I., Estruch R., Covas M.I., Fitó M., Salas-Salvadó J., Martinez-González M.A., Aros F., Lamuela-Raventós R.M. (2014). Plasma fatty acid composition, estimated desaturase activities, and their relation with the metabolic syndrome in a population at high risk of cardiovascular disease. Clin. Nutr..

[69] Von Roemeling C.A., Marlow L.A., Wei J.J., Cooper S.J., Caulfield T.R., Wu K., Tan W.W., Copland J.A. (2013). Stearoyl-CoA desaturase 1 is a novel molecular therapeutic target for clear cell renal cell carcino ma. Clin. Cancer Res..

[70] Kolanowski W. (2007). Long chain polyunsaturated omega-3 fatty acids and their role in reducing the risk of life-style related diseases. Bromat. Chem. Toksykol..

[71] Turner T.D., Jensen J., Jessica L., Pilfold J., Prema D., Donkor K.K., Cinel B., Thompson D.J., Dugan M.E.R., Church J.S. (2015). Comparison of fatty acids in beef tissues from conventional, organic and natural feeding systems in western Canada. Can. J. Anim. Sci..

[72] Webb E.C., O’Neill H.A. (2008). The animal fat paradox and meat quality. Meat Sci..

[73] Simopoulos A.P. (2011). Evolutionary aspects of diet. The omega-6/omega-3 ratio and the brain. Mol. Neurobiol..

[74] Kim W., Khan N.A., McMurray D.N., Prior I.A., Wang N., Chapkin R.S. (2010). Regulatory activity of polyunsaturated fatty acids in T-cell signaling. Prog. Lipid Res..

[75] Cichosz G., Czeczot H. (2012). Trans fatty acids in the human diet. Bromat. Chem. Toksykol..

[76] Bałasińska B., Jank M., Kulasek G. (2010). Properties and the role of polyunsaturated fatty acids in health protection of human and animal. Życie Wet..

[77] Li X., Bi X., Wang S., Zhang Z., Li F., Zhao A.Z. (2019). Therapeutic potential of n-3 polyunsaturated fatty acids in human autoimmune diseases. Front. Immunol..

[78] Kritchevsky D., Tepper S.A., Wright S., Czarnecki S.K., Wilson T.A., Nicolosi R.J. (2004). Conjugated linoleic acid isomer effects in atherosclerosis: Growth and regression of lesions. Lipids.

[79] Benjamin S., Prakasan P., Sreedharan S., Wright A.D., Spener F. (2015). Pros and cons of CLA consumption: An insight from clinical evidences. Nutr. Metab..

[80] Pogorzelska-Nowicka E., Atanasov A.G., Horbańczuk J., Wierzbicka A. (2018). Bioactive compounds in functional meat products. Molecules.

[81] Tocci R., Sargentini C. (2020). Meat quality of Maremmana young bulls. Acta Sci. Anim. Sci..

[82] Hmso U. (1994). Nutritional Aspects of Cardiovascular Disease (Report on Health and Social Subjects No. 46).

[83] Harris W.S. (2018). The omega-6:omega-3 ratio: A critical appraisal and possible successor. Prostaglandins Leukot. Essent. Fatty Acids.

[84] Popova T., Ignatova M., Petkov E., Stanišić N. (2016). Difference in fatty acid composition and related nutritional indices of meat between two lines of slow-growing chickens slaughtered at different ages. Arch. Anim. Breed..

[85] Švrčula V., Košinová K., Okrouhlá M., Chodová D., Hart V. (2019). The effect of sex on meat quality of fallow deer (*Dama dama*) from the farm located in the Middle Bohemia. Ital. J. Anim. Sci..

[86] Valfre F., Caprino F., Turchini G.M. (2003). The health benefit of sea food. Vet. Res. Commun..

[87] Kilar J. (2020). Zasoby zwierząt łownych w Polsce w aspekcie bazy surowcowej przetwórstwa mięsnego. Ochrona Środowiska Produkcji Rolniczej.

